# Reconciling the business-care paradox: a Q study of veterinarians' professional identity formation

**DOI:** 10.3389/fvets.2026.1873736

**Published:** 2026-06-25

**Authors:** Qianwen Joyce Yu, Si-Jia Sun

**Affiliations:** 1 School of International Studies, Soochow University, Suzhou, Jiangsu, China; 2 Division of Cardiology, Department of Medicine, The First Affiliated Hospital of Soochow University, Soochow University, Suzhou, Jiangsu, China

**Keywords:** emotional labor, paradox, professional identity, veterinary education, veterinary medicine

## Abstract

**Introduction:**

Veterinary medicine is shaped by the business-care paradox, a persistent tension between commercial sustainability and ethical care commitments. Emotional labor is central to how practitioners navigate this paradox, yet the subjective processes through which they construct and sustain professional identity amid these competing demands have received little empirical attention from a subjectivity standpoint. Existing studies illuminate how emotions mediate this paradox yet stop short of examining the distinct identity pathways that practitioners adopt.

**Methods:**

Employing Q-methodology, 30 small-animal veterinarians in employee roles across private practices in southeastern China sorted 42 statements. The statements were developed around four core mechanisms of emotion-mediated paradox navigation: emotional salience, flexible emotional labor, emotional traces, and ongoing learning. Principal component analysis with varimax rotation identified shared subjectivity patterns, while semi-structured post-sorting interviews elicited rationales for extreme placements, strengthening interpretive validity.

**Results:**

A three-factor solution explained 58% of variance, revealing three distinct identity trajectories. The *pragmatic service provider* reframes business decisions as necessary for sustainability, prioritizing clinic viability, and employs emotional detachment as a protective strategy. The *conflicted caregiver* experiences moral distress and identity dissonance when financial constraints limit care, feeling torn between ideal and feasible treatment. The *resilient integrator* treats constraints as parameters for creative problem-solving, drawing on adaptive learning while sustaining core ethical commitments. These viewpoints represent qualitatively distinct modes of emotional labor engagement.

**Discussion:**

The findings reveal three distinct modes of emotional labor engagement, each reflecting a different pathway of professional identity construction. For veterinary educators, these findings highlight the need for differentiated support strategies, each tailored to a distinct identity trajectory. Spanning moral distress mitigation to resilience cultivation, such strategies are essential for sustaining both practitioner wellbeing and care quality.

## 1 Introduction

Veterinary medicine is shaped by the business-care paradox, a pervasive tension between commercial sustainability and ethical care commitments that permeates daily clinical decision-making (1, 2). This tension exposes practitioners to significant emotional challenges including chronic stress, burnout, and moral distress, even as successful clinical outcomes and the human-animal bond generate profound fulfillment (3, 4). Previous studies have documented the emotional intensity of specific veterinary scenarios such as euthanasia, adverse outcomes, and client interactions constrained by financial limitations (5–7). Large-scale surveys further confirm that moral distress is a pervasive experience when financial constraints or client preferences prevent veterinarians from delivering what they consider appropriate care, and that the majority of practitioners enter the profession with little to no training in navigating these ethical conflicts (8, 9). Recent work reframes these experiences not as isolated stressors but as the cumulative impact of systemic occupational trauma, underscoring the need for institutional rather than solely individual solutions (10). These studies collectively establish that navigating such situations demands not only clinical proficiency but also sustained emotional regulation.

While veterinary medicine represents a distinct professional context, its core tension renders it a particularly illuminating case for health professions research. As Pradies (2) observes, the business-care paradox manifests with striking immediacy in veterinary practice, where practitioners must directly negotiate care commitments with clients' immediate financial constraints in ways that institutional structures in other health professions tend to buffer. This makes veterinary medicine a revelatory setting for examining how professionals construct and sustain occupational identities when faced with competing institutional logics, and how emotional labor mediates the relationship between these paradoxes and identity formation (11).

The concept of emotional labor, defined as the conscious management of internal feelings and outward emotional expressions to meet occupational expectations, provides a critical lens for understanding this aspect of veterinary work (11). Hochschild distinguishes between surface acting, in which individuals manage outward expressions without modifying internal feelings, and deep acting, in which they consciously adjust internal emotional states to align with expected displays (11). Within healthcare research, emotional labor is recognized as a mediator between institutional demands and professional conduct (12). However, its application within veterinary medicine remains comparatively underdeveloped. A nascent body of work indicates that how veterinarians manage emotional labor is deeply intertwined with processes of professional identity construction, a relationship further complicated by the context-specific nature of the business-care paradox (1, 2, 13). This paradox generates conflicting role expectations that may produce identity dissonance, a persistent sense of incongruence between practitioners' internal ethical commitments and the external commercial pressures that structure their work (13).

The emotion-mediated paradox navigation framework (2) provides a valuable theoretical account of this process by positing four core mechanisms. *Emotional salience* captures how personal connections to tensions render the paradox subjectively meaningful. *Flexible emotional labor* describes the intuitive oscillation between competing demands. *Emotional traces* refer to the lingering feelings following a paradoxical encounter. *Ongoing learning* denotes how these traces inform future navigation strategies. Together, these four mechanisms constitute an integrated process through which emotional labor mediates the relationship between the business-care paradox and the professional identities that practitioners construct. While this framework effectively maps the emotional dynamics of paradox navigation, it stops short of illuminating how this process is subjectively enacted and what distinct identity templates practitioners adopt when navigating the same structural paradox.

Previous qualitative studies, though rich in contextual detail, have yet to identify the shared subjectivity patterns shaping professional identity in this contested domain (2, 13). Understanding these subjective standpoints is crucial, since they are likely to govern how individuals experience moral distress, deploy emotional labor, and sustain their professional roles over time. This gap carries direct implications for veterinary education. Without a detailed map of the subjective landscapes that practitioners inhabit, educational programs risk offering generalized ethics and communication training that fails to address the distinct ways in which graduates interpret and respond to the paradox in practice.

**Figure 1:**
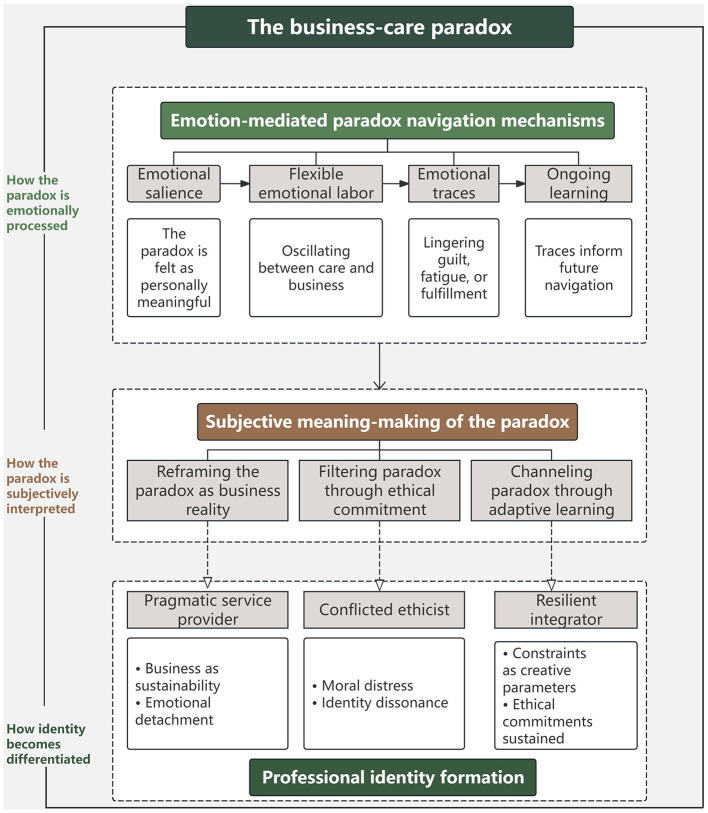
Subjectivity-differentiated model of paradox navigation and professional identity formation.

This study therefore asks not whether veterinarians experience the business-care paradox, but how they subjectively construct their professional identity through it. Employing Q-methodology, an integrative approach designed to investigate subjectivity (14), this study aims to identify shared perspectives among Chinese veterinarians as they negotiate the business-care paradox, thereby complementing a literature that has primarily drawn on Western contexts. The Q-set development was guided by the four mechanisms of Pradies (2)'s framework. Figure 1 presents the subjectivity-differentiated model that underpins this study, illustrating how the same paradox navigation mechanisms may, through distinct modes of subjective meaning-making, give rise to divergent professional identity trajectories. By mapping these distinct subjectivities, the study seeks to establish an empirical basis for designing targeted educational interventions that foster resilient professional identity formation in veterinary medicine.

## 2 Methods

### 2.1 Research design

Q-methodology is a research approach that integrates by-person factor analysis with qualitative interpretation to systematically investigate subjectivity (14). Unlike conventional survey methods that require participants to respond to researcher-defined categories, Q-methodology allows participants to articulate their perspectives through a structured ranking procedure, making it particularly suited to uncovering shared viewpoints, including minority or contested positions, within a defined group. In this study, participants sorted a set of statements along a continuum of agreement, converting individual subjective judgments into intercorrelated factor arrays amenable to both statistical analysis and qualitative interpretation (15). This approach thus integrates quantitative rigor with qualitative depth to identify distinct shared viewpoints.

### 2.2 Concourse development and Q-Set construction

The concourse was developed from two sources. First, we reviewed literature on veterinarians' emotional labor, professional identity, and paradox navigation to identify relevant concepts and expressions (2, 5, 13, 16). This process was guided by the four mechanisms of the emotion-mediated paradox navigation framework: *emotional salience, flexible emotional labor, emotional traces*, and *ongoing learning* (2). These mechanisms provided the theoretical structure for conceptual sampling. Second, we drew upon transcripts from a separate set of 15 semi-structured qualitative interviews with veterinarians purposively sampled from small-animal private practices in southeastern China (conducted between September and November 2023). These interviews, lasting 40–60 min each, elicited narratives about challenging cases, financially constrained client interactions, ethical dilemmas, and coping strategies. The interviewees varied in gender, years of experience, and practice type, and none were included in the subsequent Q-sort sample, thereby safeguarding the independence of the subjective viewpoints subsequently captured.

Statements from both sources were screened by the research team from an initial pool of 58. Each statement was evaluated for clarity, redundancy, and relevance to both the theoretical dimensions and the lived experience of veterinarians. This process yielded a final Q-set of 42 statements. To ensure theoretical coherence, both authors independently mapped each statement to one of the four framework mechanisms based on whether its central theme reflected *emotional salience, flexible emotional labor, emotional traces*, or *ongoing learning* as defined by Pradies (2). In cases where a statement touched upon more than one mechanism, it was assigned to the dimension most prominently reflected in its wording. Initial independent mapping showed high agreement; the few discrepancies were resolved through discussion. The final mapping is presented alongside the full Q-set in Supplementary Appendix A.

### 2.3 P sampling

Data were collected between March and August 2024 by the first author in three cities in southeastern China (Guangzhou, Shenzhen, and Zhuhai). Consistent with Q-methodology, which prioritizes diversity of subjective viewpoints over statistical representativeness, a purposive sampling strategy was employed. To ensure a homogeneous context in which the business-care paradox is most salient, two criteria were applied. First, participation was limited to veterinarians in employee roles, excluding practice owners and partners, so that the study captured practitioners who enact rather than set organizational business policies. Second, all participants were recruited from private clinics specializing in small-animal general practice, ensuring that paradoxical tensions arose from a common set of clinical and financial challenges. Participants were recruited through professional veterinary networks and snowball sampling, with initial contacts established via regional veterinary associations. Thirty veterinarians meeting these criteria were recruited from private practices in southeastern China. Within this scope, the P-set maintained diversity across gender and years of clinical experience to capture a rich variety of viewpoints within the defined parameters. Participant demographics are summarized in Table 1.

**Table 1:** Participants' demographic information.

Participant ID	Gender	Age range	Years of experience
VET01	F	25–29	5
VET02	M	30–34	7
VET03	F	35–39	9
VET04	M	25–29	4
VET05	M	30–34	6
VET06	F	40–44	14
VET07	M	35–39	8
VET08	F	25–29	3
VET09	F	30–34	6
VET10	M	35–39	10
VET11	M	25–29	4
VET12	F	30–34	7
VET13	F	40–44	15
VET14	M	35–39	11
VET15	F	30–34	8
VET16	M	25–29	5
VET17	F	35–39	9
VET18	M	30–34	6
VET19	F	40–44	13
VET20	M	35–39	12
VET21	M	25–29	4
VET22	F	30–34	7
VET23	F	35–39	8
VET24	M	40–44	15
VET25	F	25–29	5
VET26	M	30–34	6
VET27	M	35–39	10
VET28	F	30–34	8
VET29	M	25–29	3
VET30	F	35–39	11

### 2.4 Q sorting

Each participant was presented with the 42 statements in randomized order and instructed to sort them along a quasi-normal distribution grid ranging from −5 (most disagree) to +5 (most agree). Participants ranked the statements according to their relative agreement, with no two statements occupying the same position. They were explicitly instructed to interpret each statement based on their own subjective understanding.

Following the sorting task, a semi-structured interview was conducted to elicit participants' rationales for placing statements at the extremes of the distribution. Interviews lasted approximately 15–25 min, were audio-recorded with participants' consent, and were transcribed verbatim. The interview guide covered three core areas: the reasoning behind the most strongly agreed and disagreed statements, how these choices reflected professional experiences with the business-care paradox, and any statements that were difficult to place or appeared to conflict with others (Supplementary Appendix C). By the final interviews, participants' explanations were consistently reinforcing the emerging factor interpretations, with no substantively new rationales arising, indicating sufficient depth for interpretive purposes. The interview data thus enhanced the credibility and richness of these interpretations.

### 2.5 Data analysis

Completed Q-sorts were analyzed using PQMethod (Version 2.35). Principal component analysis was performed on the correlation matrix of all Q-sorts. Factor retention was guided by the Kaiser-Guttman criterion (eigenvalue >1.0) and by the conceptual interpretability and distinctiveness of the emergent viewpoints (14). Retained factors were subjected to Varimax rotation to produce a clearer factor structure (17). Significant factor loadings were determined using the standard error formula (SE = 1/√*N*, where *N* = 42 is the number of Q-set items); the conservative threshold of ±0.40 was therefore adopted as the criterion for a statistically significant loading (17, 18). Factors with fewer than two significantly loading Q-sorts were excluded, ensuring that each shared viewpoint was defined by more than a single individual (14). A two-factor and a four-factor solution were examined but yielded less conceptually coherent patterns or failed to meet the minimum eigenvalue criterion for all factors, supporting the retention of three factors. This procedure yielded the three-factor solution detailed in the Results section. Factor loadings for all participants are provided in Supplementary Appendix B. Factor interpretation followed the standard procedure of examining distinguishing statements and characterizing statements within each factor array, supplemented by post-sorting interview data to enhance interpretive depth (14). In Q-methodology, each factor array represents a composite viewpoint constructed from the Q-sorts of significantly loading participants. The scores assigned to each statement within a factor indicate the relative importance of that statement in defining the viewpoint, with extreme scores (e.g., +5, −5) marking the statements that most distinguish one perspective from the others. Factor interpretation focuses on these distinguishing statements alongside post-sorting interview data to build a qualitative portrait of each shared subjectivity.

Post-sorting interview data were analyzed using thematic analysis (19) to provide contextual depth for factor interpretation. The first author developed preliminary interpretations by examining factor arrays alongside interview transcripts. Theseconds author then independently reviewed the same materials while blinded to the initial interpretations and proposed alternative analyses. The authors subsequently engaged in consensus discussions to resolve interpretive differences and establish definitive labels and characteristics for each viewpoint. Factor labels were derived jointly from the distinguishing statements, factor arrays, and participants' qualitative narratives. This investigator triangulation enhanced the trustworthiness of the final factor interpretations (20).

## 3 Results

Analysis of the Q-sort data produced a three-factor solution accounting for 58% of total variance, exceeding the recommended threshold of 35% for robust interpretation of shared subjectivities (14). The factors represent constructed ideal types that capture shared patterns of subjectivity, rather than empirically discrete categories into which individual veterinarians can be exhaustively sorted. Each factor represents a qualitatively distinct mode of engaging in emotional labor and constructing professional identity. Of the 30 Q-sorts, eight loaded significantly on Factor one, seven on Factor two, and seven on Factor three. The remaining eight Q-sorts did not load significantly on any single factor, indicating that their viewpoints were not shared widely enough to constitute a distinct perspective or were confounded across factors. Factor interpretation proceeded by examining the statements ranked at the extremes of each factor array, supplemented by post-sorting interview data from defining participants. Three statements emerged as consensus items, with normalized factor scores not differing significantly across any pair of factors: transparency about treatment costs (Statement #31, +2 across all factors), the lasting sense of professional accomplishment from saving a critically ill animal (Statement #16, +3), and client gratitude as an antidote to emotional fatigue (Statement #35, +3). This indicates broad consensus that certain professional norms and intrinsically rewarding aspects of clinical work transcend the divergent identity trajectories identified here. The complete factor arrays are provided in Supplementary Appendix A.

### 3.1 Factor one: the pragmatic service provider

Factor one accounted for 24% of the study variance and was defined by eight significantly loading participants. This viewpoint is characterized by strong agreement that veterinary medicine is fundamentally a business requiring operational discipline and that emotional detachment is necessary for rational decision-making (Statement #5, +5; Statement #11, +5). It also endorses the use of medical terminology to facilitate difficult decisions and the avoidance of emotionally charged language during consultations (Statement #8, +4; Statement #13, +4). This viewpoint most strongly rejects feelings of moral failure or guilt following financially constrained euthanasia or suboptimal treatment (Statement #15, −5; Statement #6, −5).

This sorting pattern reflects a mode of paradox navigation in which emotional salience is cognitively downplayed, surface-level emotional labor predominates, and negative emotional traces are actively suppressed through reframing business decisions as necessary for sustainability rather than as ethical compromises. Consistent with this, Factor one participants rated the statement regarding desensitization to the financial aspects of life-and-death decisions positively (Statement #20, +3), while rejecting the suggestion that they lie awake thinking about animals they could not save due to cost barriers (Statement #19, −4).

Interview data reinforced a consistent focus on operational viability as the foundation for ethical practice. Two defining participants articulated this logic with notable clarity:

VET14: “We have equipment loans, staff salaries […] I can't let emotions dictate financial decisions. It's not sustainable”.

VET05: “My responsibility is to keep the clinic running. If I let every case get to me emotionally, I wouldn't last in this profession […] It's about the long-term ability to help”.

The ongoing learning dimension for this group appeared oriented toward practical skill acquisition rather than ethical reflection. They agreed that they have become more skilled at predicting client reactions to cost estimates (Statement #36, +3) and endorsed learning to set emotional boundaries as essential for long-term wellbeing (Statement #27, +3). Two further participants elaborated on how pragmatic detachment is cultivated as a deliberate professional strategy:

VET01: “At the end of the day, this is a business. If we close, nobody gets helped. That's the reality […] most idealistic graduates don't see”.

VET20: “I've learned that being pragmatic about money doesn't make you heartless […] It makes you capable of helping more animals over the long run”.

The professional identity constructed here is that of a realistic service provider for whom financial stability is a prerequisite for sustainable ethical practice. Emotional detachment is framed not as a deficit of care but as a professional necessity for enduring in the field.

It is important to note that this orientation does not imply an absence of commitment to animal welfare. Rather, it reflects a pragmatic conviction that clinical care can only be delivered sustainably within a viable business framework. In terms of identity formation, this trajectory is sustained by cognitively reframing the business-care paradox as a practical challenge rather than an ethical dilemma, thereby minimizing the emotional traces that might otherwise destabilize professional self-concept.

### 3.2 Factor two: the conflicted caregiver

Factor two accounted for 19% of the study variance and was defined by seven significantly loading participants. This viewpoint is defined by strongest agreement with statements expressing felt tension between optimal patient care and client affordability, accompanied by guilt when ideal treatment cannot be provided (Statement #3, +5; Statement #6, +5). Defining participants also strongly endorse the importance of being seen as caring and compassionate rather than merely a businessperson, and they affirm a strong personal connection to the animals they treat (Statement #2, +5; Statement #1, +4). This viewpoint most strongly rejects the notion of desensitization to financially driven life-and-death decisions and the necessity of emotional detachment (Statement #20, −5; Statement #11, −5), and disagrees with avoiding emotionally charged language during consultations (Statement #13, −4). The label “Conflicted Caregiver” is used analytically to capture a pattern of subjectivity in which practitioners frame the business-care paradox through the lens of their care commitments, experiencing financial constraints as moral burdens rather than operational realities.

This pattern illustrates a mode of paradox navigation in which emotional salience remains acutely activated and flexible emotional labor is constrained by deeply internalized ethical commitments. Emotional traces accumulate as guilt and sleepless rumination rather than being resolved or reframed through adaptive learning. Factor two participants strongly agreed that they lie awake at night thinking about animals they could not save due to cost barriers (Statement #19, +5) and found it emotionally draining to constantly justify the cost of life-saving treatments to skeptical clients (Statement #39, +5). Two defining participants described how these experiences erode their professional self-concept:

VET07: “Among the most disheartening experiences […] is performing euthanasia when treatment becomes financially unfeasible […] It compels me to question myself: do I still deserve to be called a veterinarian?”

VET16: “I lay awake at night thinking about the animals I could not save due to cost barriers. It […] erodes the very reason I entered this field”.

Critically, the ongoing learning dimension appears underdeveloped for this group. They rejected the idea that they have become desensitized over time (Statement #20, −5), and they did not endorse statements reflecting adaptive reframing, such as viewing compromises as necessary adaptations (Statement #28, −2) or focusing on animals they have helped rather than those they could not save (Statement #33, −1). The business side of practice frequently led them to question their original calling (Statement #32, +5). Two further participants elaborated on the cumulative nature of this unresolved moral distress:

VET02: “Every time I have to say ‘we could do more, but it will cost...' […] I feel like I'm betraying the oath I took”.

VET12: “People say you get used to it. I haven't. Five year in, and the guilt only accumulates […] I carry every case where money was the reason we stopped”.

The professional identity associated with this factor remains unsettled, defined by ongoing moral tension between core ethical values and the practical constraints of clinical reality. Unlike Factor one, where business logic provides a cognitive framework that resolves the paradox, Factor two participants experience the paradox as an unresolved and deeply personal moral burden. Identity formation in this trajectory is marked by a continued struggle to reconcile internal ethical commitments with external commercial pressures, a process in which the absence of effective justificatory frameworks leaves practitioners vulnerable to chronic moral distress and identity erosion.

### 3.3 Factor three: the resilient integrator

Factor three accounted for 15% of the study variance and was defined by seven significantly loading participants. This viewpoint is characterized by strongest agreement that continuous learning is key to maintaining both clinical competence and emotional resilience, and that financial constraints are parameters for creative problem-solving rather than insurmountable barriers (Statement #25, +5; Statement #38, +5). Defining participants also strongly endorse seeing themselves as educators helping clients understand the value of preventive care and building long-term trust over maximizing single-visit profit (Statement #12, +5; Statement #14, +5). This viewpoint rejects the notion that the business side of practice undermines one's calling to help animals and opposes the view that veterinary medicine is fundamentally a business (Statement #32, −4; Statement #5, −4).

This pattern reflects a mode of paradox navigation in which emotional salience is acknowledged but not overwhelming, emotional labor is deployed flexibly across care and business demands, and emotional traces of pride and accomplishment feed into ongoing learning, reinforcing adaptive strategies over time. Two defining participants described how this integrative stance was cultivated through deliberate cognitive reframing:

VET22: “I now view compromises not as failures, but as necessary adaptations within a complex system. I see financial constraints as a […] parameter within which to work creatively”.

VET03: “Early in my career I nearly burned out from the guilt. What changed was realizing that there's always another way, like, […] a cheaper drug, a staged treatment plan. I had to learn to think that way”.

Factor three participants endorsed multiple statements reflecting active, deliberate learning: mentally preparing for difficult client interactions (Statement #22, +5), reflecting on past cases to develop better cost-communication strategies (Statement #23, +5), developing a personal set of principles for business-care dilemmas (Statement #26, +4), actively seeking advice from senior colleagues (Statement #24, +4), and consciously using nonverbal cues to build rapport during cost discussions (Statement #41, +4). They also endorsed the view that this profession has taught them that perfect care is often unattainable and good-enough care is a valid goal (Statement #42, +4), suggesting a philosophical acceptance of clinical limits not evident in the other two factors. Two further participants elaborated on how this philosophical acceptance translates into daily practice:

VET29: “I have learned to mentally prepare for difficult conversations. Every client interaction […] is a chance to educate and find a path forward that respects both care and cost”.

VET06: “The business side isn't the enemy of good care. It's […] the structure within which care happens. Once I accepted that, I stopped fighting the system and started working with it”.

The professional identity here is integrative, combining the roles of healer, educator, and businessperson into a cohesive whole. Resilience in this group stems not from emotional detachment (as in Factor one) nor from unresolved moral tension (as in Factor two), but from viewing the paradox itself as a site of professional growth through adaptive learning. Identity formation in this trajectory is sustained by ongoing learning processes that transform emotionally challenging experiences into resources for professional development, enabling practitioners to maintain ethical commitment without being consumed by the tensions that destabilize the other trajectories.

## 4 Discussion

This study employed Q-methodology to map the subjective landscapes that veterinarians inhabit when navigating the pervasive business-care paradox. The identification of three distinct factors provides a detailed, empirically grounded understanding of how professional identity is constructed and negotiated through the emotional labor of managing competing demands. Our findings extend the emotion-mediated paradox navigation framework (2) by illustrating how its core mechanisms are enacted through qualitatively distinct modes of subjectivity, each representing a different pathway of identity formation.

### 4.1 Subjectivity in paradox navigation

What these trajectories reveal is that the four mechanisms do not operate uniformly. Emotional salience, for instance, registers as acute moral tension for the *conflicted caregiver* yet barely surfaces for the *pragmatic service provider*. This indicates that each mechanism is filtered through the meaning-making frameworks practitioners bring to the paradox, giving rise to divergent identity outcomes rather than a single adaptive endpoint. This subjectivity-differentiated view addresses the gap identified earlier by showing that the answer lies in the interpretive frameworks practitioners bring to the paradox. These frameworks filter how emotional salience is registered, how emotional labor is deployed, how traces are experienced, and how learning unfolds.

This divergence can be understood through the lens of professional identity formation. In their work on veterinary professional identity, Armitage-Chan and May (21) have described how practitioners who integrate contextual complexity, such as clients' financial constraints, into their sense of professional self are better able to adapt flexibly to competing demands. Those who are unable to form this connection tend to experience environmental challenges not as manageable features of practice but as persistent obstructions to their identity goals. Our findings extend this work by demonstrating that emotional labor constitutes a key mechanism through which these divergent identity trajectories are enacted and sustained.

First, the three viewpoints reveal how emotional salience functions as a differentiating mechanism that channels practitioners toward distinct identity trajectories, rather than a uniform experience. For the *conflicted caregiver*, the paradox is acutely salient, manifesting as intense moral tension that destabilizes an idealized care-centered identity. This aligns with literature documenting the profound distress veterinarians experience in ethically charged situations such as financial euthanasia (5, 22–24) and with survey findings that moral distress is both prevalent and consequential for professional wellbeing (8, 9). The prevalence of this experience is underscored by recent data showing that nearly 95% of emergency veterinarians regularly encounter situations in which clients' financial limitations prevent recommended treatment, with over 40% of early-career practitioners identifying such cases as among the most distressing aspects of their work (25). The moral tension characterizing this trajectory can be understood as a conflict between personal moral convictions and external obstacles, a dynamic captured by the question of whether one is acting as a veterinarian or an economist (26). Similarly, early-career veterinarians are also regarded as oscillating between a socially dominant diagnosis-focused identity and a locally valued relational identity, unable to commit consistently to either and experiencing a persistent sense of being the wrong kind of professional (27). In the *conflicted caregiver*, this confusion manifests as an unresolved tension between internalized care commitments and the commercial realities of practice, with practitioners questioning the legitimacy of their professional identity. In contrast, the *pragmatic service provider* has reframed this salience through cognitive distancing, adopting an identity centered on a commercial logic. This supports the notion that emotional labor involves complex processes of cognitive reframing to align with organizational demands (4, 11, 28, 29), particularly in veterinary contexts where business norms are foregrounded (1, 2, 30). The *resilient integrator* acknowledges the salience of the paradox without being overwhelmed by it, using it as an impetus for adaptive learning. This spectrum of responses contributes to paradox literature in showing that emotional salience initiates divergent processes of identity work (2, 6, 13, 31).

Second, the three trajectories illuminate distinct patterns of flexible emotional labor. The *pragmatic service provider* relies on surface acting (11) and justifications rooted in business norms, enacting a professional identity organized around business viability. This orientation is not simply an individual coping strategy; it reflects the structural realities of private veterinary practice, particularly in competitive urban markets. As previous research documents, high rental costs, dense clinic distribution, and client-driven competitive pressures create an environment in which business sustainability is a pressing daily concern (32). The finding that many veterinarians receive no business training during their education further compounds this pressure, leaving practitioners to negotiate the business-care paradox without adequate preparation. The *conflicted caregiver*, while engaging in deep acting (11), often lacks effective justificatory frameworks to resolve their distress, leaving their identity in a state of dissonance that reflects a strong personal connection to paradoxical tensions (2, 33, 34). The *resilient integrator* exemplifies flexible emotional labor, intuitively oscillating between competing demands through internalized practices that harmonize competing logics (35–37). This finding advances the understanding that emotional labor is fundamentally constitutive of veterinary professional identity, rather than merely a set of behaviors (13, 38, 39).

The *emotional traces* and *ongoing learning* mechanisms further clarify the longitudinal dimension of identity construction. The negative traces of fatigue and guilt reported by the *conflicted caregiver* risk leading to burnout and identity erosion, consistent with previous findings about veterinary mental health (22, 24). Notably, McCobb et al. (25) found that veterinarians with more than 16 years of experience were significantly less likely to report severe distress in response to financial limitations than those with five or fewer years of experience. This pattern suggests that, for some practitioners, ongoing exposure to the paradox may facilitate the development of adaptive strategies that mitigate its emotional impact over time. Conversely, the positive traces of energy and accomplishment fuel the *resilient integrator*'s identity, reinforcing their sense of purpose and professional competence. The transition from reactive coping to proactive regulation represents a move from merely experiencing emotions to orchestrating them for professional growth, embodying the concept of ongoing learning where emotional traces inform future navigation strategies (2, 40, 41). Importantly, this learning process does not occur in a vacuum; as recent trauma-informed perspectives emphasize, practitioners' capacity to learn from emotional experience depends substantially on the organizational environments in which they work (10). The negative emotional traces reported by the *conflicted caregiver* may be further compounded by professional cultural narratives that position the client as an obstacle to quality care, reinforcing a context-inappropriate identity that excludes the relational dimensions of practice (27). This process of learning from emotional experiences helps practitioners establish workable barriers against emotional exhaustion while maintaining professional engagement (13, 42, 43).

Overall, this study provides a significant theoretical extension of emotional labor in paradox navigation by elucidating the role of subjectivity in professional identity construction. The identification of three distinct viewpoints reveals that the framework's core mechanisms are not uniform processes but are instead enacted through distinctly different subjective lenses, giving rise to divergent identity trajectories. It bears emphasis that none of these three identity trajectories is inherently superior; each represents a coherent mode of navigating the paradox that may be more or less adaptive depending on the practitioner's specific clinical context, career stage, and available institutional support. These findings align with the recognition that a single adaptive endpoint does not define professional success in veterinary medicine (21) and reinforce the observation that, for many practitioners, the core ethical challenge lies not in resolving open ethical dilemmas but in managing the tension between personal moral convictions and external obstacles (26). Thus, the study shows how veterinarians differentially interpret and internalize paradoxical tensions to build, maintain, or struggle with their professional identities, foregrounding the centrality of subjectivity in paradox theory.

### 4.2 Implications for veterinary education

The identification of these three shared viewpoints carries direct implications for veterinary education, moving it beyond standardized ethics and communication training. These findings suggest that veterinary curricula should prepare students for the full spectrum of identity tensions they may encounter in practice, rather than offering generic ethics training that assumes a uniform experience of the business-care paradox. For students who experience moral distress when clinical decisions are constrained by financial realities, reflective writing, narrative medicine exercises, and accessible mental health resources can provide structured opportunities to process these tensions before they entrench into burnout (5, 22, 24, 38, 44). The finding that a considerable number of practicing veterinarians receive little to no training in navigating ethical conflicts (8, 9) underscores the urgency of this curricular gap. At the same time, students who gravitate toward framing clinical work primarily through commercial logic may benefit from ethics simulations that illustrate the long-term consequences of purely utilitarian decisions and from communication training that demonstrates how trust, rather than transactional efficiency, sustains practice viability (1, 30). The Resilient Integrator trajectory further suggests that these diverse coping strategies need not compete. Through case-based learning in which students practice generating solutions that honor both care and business imperatives, pedagogy that explicitly teaches a paradox mindset may help cultivate the adaptive flexibility that characterizes this trajectory. Rather than steering students toward a particular identity pathway, the aim is to equip all graduates with a repertoire of reflective and emotional skills broad enough to navigate the paradox in whichever form it manifests in their professional lives.

Beyond addressing identity tensions, the findings further underscore that emotional labor skills can be developed through structured training integration (13, 23, 33). Veterinary curricula should incorporate simulated client interactions using standardized protocols, allowing students to practice delivering costly treatment plans and discussing euthanasia in safe environments with focused feedback on communicating in emotionally charged situations. Explicit training in emotional self-awareness should empower students to recognize their emotional triggers and default coping styles, enabling conscious response selection rather than automated habits. Fundamental to these skills is normalizing the business-care paradox through open discussion and shared vocabulary for the complex emotions it evokes. As trauma-informed approaches to veterinary practice suggest, reframing these struggles as central to professional experience rather than as personal failing can reduce shame and isolation while building a foundation for sustainable practice (4, 10, 28).

### 4.3 Strengths, limitations, and future directions

A key strength of this study lies in its use of Q-methodology, an integrative approach that combines by-person factor analysis with qualitative interpretation to systematically examine the formation of professional identity. This design offers insights into how veterinarians navigate paradoxical tensions through emotional labor that would be difficult to capture through conventional survey or interview methods alone. Nevertheless, several limitations should be considered. First, while Q-methodology effectively identifies shared viewpoints, it captures cross-sectional perspectives rather than longitudinal identity development. The pathways described here represent distinct subjective positions at one point in time; how practitioners move between or evolve through these pathways remains an open question. Second, the sample was drawn exclusively from private small-animal practices in southeastern China. While this focus strengthens the study's internal coherence, it raises questions about the generalizability of the identified identity trajectories. As Chan et al. (32) note, the urbanized, competitive business environment of Chinese cities creates specific pressures that may not be present in other cultural or economic settings. The correspondence between some of our findings and those from North American and European contexts (9, 26) suggests a degree of universality to the business-care paradox, but the specific expression and prevalence of the three identity trajectories likely varies across settings. Comparative Q-studies across diverse healthcare systems and cultural contexts would help distinguish universal professional tensions from context-specific influences.

These limitations open several avenues for future research. First, the three-factor solution accounted for 58% of total variance. While this exceeds the recommended threshold of 35% for Q-methodological studies (14), 42% of variance remained unexplained, and 8 of 30 participants did not load significantly on any factor. These unloaded Q-sorts may reflect genuinely idiosyncratic perspectives that were not shared widely enough to constitute additional factors, or they may signal aspects of the business-care paradox that the Q-set did not adequately capture. Future research employing an expanded or differently structured concourse could examine whether additional shared viewpoints emerge. Second, longitudinal qualitative studies tracking veterinary professionals from training into practice could clarify how these identity trajectories develop, stabilize, or shift over time. Comparative Q-studies across diverse healthcare systems and cultural contexts would help distinguish universal professional tensions from context-specific influences. Finally, research integrating individual-level identity findings with organizational-level interventions remains an important direction. As trauma-informed perspectives argue, sustainable practitioner wellbeing requires not only individual resilience but also institutional structures that normalize ethical complexity and provide systematic support (10). Future work could examine how practice management policies, mentorship structures, and professional culture shape the availability and viability of different identity pathways identified in this study.

## 5 Conclusion

This study demonstrates that veterinarians' experience of the business-care paradox is characterized by distinct shared subjectivities, representing three qualitatively different pathways of professional identity construction. By mapping these identity trajectories, the study shows that the business-care paradox does not produce a single adaptive response. Instead, it is navigated through qualitatively different modes of emotional labor, each carrying distinct implications for practitioner wellbeing. The subjectivity-differentiated model underpinning this study provides an empirical basis for rethinking how emotional labor and identity formation are addressed in veterinary education. Moving beyond generic communication and ethics training, educational programs might intentionally develop differentiated support strategies, each tailored to a distinct identity trajectory, spanning moral distress mitigation to resilience cultivation. Such development could enable future practitioners to navigate a profession defined by paradox while sustaining both ethical commitment and personal wellbeing.

## Data Availability

The raw data supporting the conclusions of this article will be made available by the authors, without undue reservation.
