# Supplementary material for: Reconciling the business-care paradox: a Q study of veterinarians' professional identity formation
Source: Front Vet Sci. 2026 Jun 25;13:1873736. doi: 10.3389/fvets.2026.1873736 (PMC13345845; doi:10.3389/fvets.2026.1873736)
Supplement: Supplementary file 1 [file Table_1.DOCX]

**Appendix A. Statements and factor arrays**

| **No.** | **Dimension** | **Statement** | **Factor** | | |
| --- | --- | --- | --- | --- | --- |
|  |  |  | **1** | **2** | **3** |
| 1 | Emotional Salience | I feel a strong personal connection to the animals I treat, which makes financial discussions emotionally charged. | −1 | +4 | +1 |
| 2 | Emotional Salience | It is important to me that clients see me as caring and compassionate, not just as a businessperson. | −3 | +5 | 0 |
| 3 | Emotional Salience | I often feel torn between what is best for the animal and what the client can afford. | −2 | +5 | +3 |
| 4 | Emotional Salience | Seeing a pet recover against the odds is what makes this profession meaningful to me. | +3 | +4 | +3 |
| 5 | Emotional Salience | I believe veterinary medicine is fundamentally a business, and we must operate accordingly to survive. | +5 | −4 | −4 |
| 6 | Emotional Salience | I feel guilty when I cannot provide the ideal treatment due to a client’s financial constraints. | −5 | +5 | +1 |
| 7 | Emotional Salience | The emotional bond between a pet and its owner is a central factor in my clinical decisions. | −1 | +4 | 0 |
| 8 | Flexible Emotional Labor | I use medical terminology to make difficult decisions easier for clients to accept. | +4 | −2 | +2 |
| 9 | Flexible Emotional Labor | I adjust my communication style based on the client’s educational background and financial situation. | +1 | 0 | +4 |
| 10 | Flexible Emotional Labor | I sometimes offer discounted services or payment plans to clients who are genuinely in need. | −3 | +3 | +3 |
| 11 | Flexible Emotional Labor | It is necessary to maintain emotional detachment to make rational business decisions. | +5 | −5 | −1 |
| 12 | Flexible Emotional Labor | I see myself as an educator, helping clients understand the value of preventive care. | +2 | +1 | +5 |
| 13 | Flexible Emotional Labor | I avoid using emotionally charged words like “death” or “suffering” during consultations. | +4 | −4 | 0 |
| 14 | Flexible Emotional Labor | Building long-term trust with clients is more important than maximizing profit from a single visit. | +2 | +3 | +5 |
| 15 | Emotional Traces | After performing euthanasia for financial reasons, I often feel a sense of moral failure. | −5 | +5 | 0 |
| 16 | Emotional Traces | Successfully saving a critically ill animal gives me a lasting sense of professional accomplishment. | +3 | +3 | +3 |
| 17 | Emotional Traces | I feel frustrated when clients prioritize cost over the well-being of their pet. | 0 | +1 | 0 |
| 18 | Emotional Traces | The gratitude of a client after a successful treatment reinforces my commitment to this profession. | +2 | +3 | +3 |
| 19 | Emotional Traces | I sometimes lie awake at night thinking about the animals I could not save due to cost barriers. | −4 | +5 | −1 |
| 20 | Emotional Traces | Over time, I have become desensitized to the financial aspects of life-and-death decisions. | +3 | −5 | −3 |
| 21 | Emotional Traces | Seeing a pet recover and reunite with its owner makes all the challenges of this job worthwhile. | +3 | +4 | +4 |
| 22 | Ongoing Learning | I have learned to mentally prepare myself for difficult client interactions before they happen. | +1 | +1 | +5 |
| 23 | Ongoing Learning | Reflection on past cases has helped me develop better strategies for discussing costs with clients. | +1 | +1 | +5 |
| 24 | Ongoing Learning | I actively seek advice from senior colleagues on how to handle emotionally and financially difficult cases. | 0 | +2 | +4 |
| 25 | Ongoing Learning | I believe continuous learning is key to maintaining both clinical competence and emotional resilience. | +1 | +1 | +5 |
| 26 | Ongoing Learning | I have developed a personal set of principles to guide me when facing business-care dilemmas. | +1 | 0 | +4 |
| 27 | Ongoing Learning | Learning to set emotional boundaries has been essential for my long-term well-being in this profession. | +3 | −1 | +3 |
| 28 | Ongoing Learning | I now view compromises not as failures, but as necessary adaptations within a complex system. | +2 | −2 | +5 |
| 29 | Emotional Salience | The pressure to generate revenue sometimes conflicts with my ethical duty to provide unbiased advice. | −1 | +4 | −2 |
| 30 | Emotional Traces | I feel a sense of pride when I can find a creative solution that balances care and cost effectively. | +2 | +3 | +4 |
| 31 | Flexible Emotional Labor | I think it's important to be transparent with clients about costs from the beginning of treatment. | +2 | +2 | +2 |
| 32 | Emotional Salience | The business side of veterinary practice often makes me question my original calling to help animals. | +1 | +5 | −4 |
| 33 | Ongoing Learning | I try to focus on the animals I have helped rather than those I could not save due to financial limits. | +3 | −1 | +3 |
| 34 | Flexible Emotional Labor | I believe that my role includes protecting clients from financial hardship as much as protecting their pets’ health. | −2 | +4 | +2 |
| 35 | Emotional Traces | Experiencing client gratitude is a powerful antidote to the emotional fatigue of this job. | +3 | +3 | +3 |
| 36 | Ongoing Learning | I have become more skilled over time at predicting how clients will react to cost estimates. | +3 | 0 | +4 |
| 37 | Emotional Traces | The emotional high from a successful case can sustain me through several difficult ones. | +1 | +3 | +4 |
| 38 | Ongoing Learning | I see financial constraints not as a barrier, but as a parameter within which to work creatively. | 0 | +1 | +5 |
| 39 | Emotional Traces | It is emotionally draining to constantly justify the cost of life-saving treatments to skeptical clients. | −2 | +5 | −1 |
| 40 | Emotional Salience | My professional identity is more that of a scientist-healer than a businessperson. | −4 | +3 | 0 |
| 41 | Flexible Emotional Labor | I consciously use nonverbal cues to build rapport and trust during cost discussions. | +1 | +1 | +4 |
| 42 | Ongoing Learning | This profession has taught me that perfect care is often unattainable, and that good enough care is a valid goal. | 0 | −3 | +4 |

**Appendix B. Factor loadings**

| **Participant** | **Factor loading score** | | |
| --- | --- | --- | --- |
|  | **F1** | **F2** | **F3** |
| VET01 | 0.72* | 0.21 | 0.15 |
| VET02 | 0.18 | 0.65* | 0.09 |
| VET03 | 0.25 | 0.14 | 0.81* |
| VET04 | 0.61* | 0.32 | 0.05 |
| VET05 | 0.53* | 0.28 | 0.22 |
| VET06 | 0.11 | 0.37 | 0.69* |
| VET07 | 0.29 | 0.78* | 0.11 |
| VET08 | 0.34 | 0.58* | 0.17 |
| VET09 | 0.31 | 0.49* | 0.25 |
| VET10 | 0.38 | 0.35 | 0.31 |
| VET11 | 0.45* | 0.39 | 0.12 |
| VET12 | 0.22 | 0.67* | 0.18 |
| VET13 | 0.17 | 0.24 | 0.76* |
| VET14 | 0.70* | 0.15 | 0.19 |
| VET15 | 0.33 | 0.33 | 0.38 |
| VET16 | 0.26 | 0.71* | 0.23 |
| VET17 | 0.59* | 0.22 | 0.27 |
| VET18 | 0.37 | 0.36 | 0.29 |
| VET19 | 0.20 | 0.25 | 0.63* |
| VET20 | 0.64* | 0.20 | 0.22 |
| VET21 | 0.39 | 0.39 | 0.22 |
| VET22 | 0.23 | 0.30 | 0.59* |
| VET23 | 0.35 | 0.32 | 0.33 |
| VET24 | 0.56* | 0.25 | 0.31 |
| VET25 | 0.31 | 0.36 | 0.35 |
| VET26 | 0.28 | 0.62* | 0.20 |
| VET27 | 0.36 | 0.31 | 0.37 |
| VET28 | 0.29 | 0.34 | 0.39 |
| VET29 | 0.15 | 0.28 | 0.55* |
| VET30 | 0.22 | 0.31 | 0.63* |

Note. Factor values indicate statistical significance (*p* < .01)

Appendix C. Interview Guide

1. I would like to understand your reasoning for the statements you placed at the two ends of the sorting grid.

- You placed this statement at [+5 / most agree]. Could you tell me why you agree with it so strongly?
- You placed this statement at [−5 / most disagree]. Could you explain why you disagree with it to this extent?
- Can you recall a specific experience from your practice that illustrates this position?

2. Looking at the statements you ranked as most strongly agreed and most strongly disagreed, how do these choices reflect your day-to-day experience of balancing business demands with patient care?

3. Have there been particular cases or client interactions that shaped how you think about these tensions?

4. Were there any statements that you found particularly difficult to place on the grid? If so, which ones, and what made the decision challenging?

5. Did any statements seem to conflict with each other, or did you feel that your position on certain issues could not be fully expressed within the sorting framework?

6. Is there anything else about your experience of the business-care tension in veterinary practice that you feel these statements did not capture, but that you would like to add?
